# The power of a patient's story: a practice in cultural humility

**DOI:** 10.3389/fgwh.2025.1498385

**Published:** 2025-03-11

**Authors:** Randall B. Schmidt

**Affiliations:** School of Medicine, University of Kansas Medical Center, Kansas City, KS, United States

**Keywords:** cultural humility, cultural competency, obstetrics, cesarean section, maternal request, emigrants and immigrants, refugees

## Abstract

As the United States grows increasingly diverse, healthcare providers will encounter changing patient populations. In obstetrics, patients often come with personal delivery priorities shaped by different life experiences, cultural expectations and personal perspectives, which may differ from those of their provider. Invariably, cultural conflicts can occur, especially when patient and provider priorities do not align. This article shares the story of a Congolese refugee mother within an urban metro area who faced such conflict when delivery preferences could not be accommodated. Highlighting communal apprehension to Cesarean delivery within the Congolese community, this piece also emphasizes how obstetrics providers can improve care for culturally diverse patients. In addition, this piece also discusses an American College of Obstetricians and Gynecologists (ACOG) committee opinion, cultural humility and its incorporation into future curriculum for medical education, and the author's personal reflection of this story's impact.

## 1 Introduction

During the summer of 2022, I had the opportunity to lead a project in the medical humanities exploring health barriers Congolese refugee mothers experience during pregnancy. Knowing that the United States (U.S.) is becoming increasingly diverse and that foreign-born, non-white people experience many health disparities, including higher maternal morbidity and mortality, I wished to learn what they viewed as important and challenging during their pregnancies (1, 2).

Recognizing the limitations of written and spoken communication, I made the decision to use photovoice – a participatory visual method that uses photographs to share stories and perspectives across cultures (3). Photovoice was originally developed to help participants reflect on communal strengths and weaknesses and to promote cultural dialogue by discussing photos (4). Photovoice has been shown to be effective at gathering qualitative data from marginalized perspectives (4, 5). A 2021 study by McMorrow and Saksena successfully used photovoice to gain a better understanding of structural barriers Congolese refugees face in food security (6). Similar studies have also used photovoice to learn from under-represented maternal perspectives of their healthcare, involving rural black women from North Carolina and Uganda (7, 8).

For this study participants received a camera with the open-ended prompt to take photos to share of their experience when encountering the U.S. health system throughout their pregnancy. After a week of photos, I interviewed each participant in Swahili^1^, using the photos as our guide and starting each discussion with “Niambie hadithi kuhusu picha hii” (Tell me the story behind this picture). Interviews were then transcribed and translated into English for thematic analysis. Throughout analysis, many themes were present, including transportation difficulties and language barriers. However, there was an overwhelmingly clear theme: apprehension and mistrust around Cesarean deliveries. In combination with photovoice, a more traditional case report methodology was utilized that included provider interviews and referencing the medical records to build a more complete picture of the difficulties these patients experienced within the healthcare system. I would like to tell the story of KN, who bravely told me about her birth experience in America.^2^

## 2 KN's story

KN was a G3P3 in her mid 30s who joined a growing East African refugee community in a large urban metro area in the early 2010s. Her obstetric history included a previous vaginal birth in East Africa, a Cesarean delivery in the U.S, and gestational diabetes. During the interview, I let her photographs and the topics represented in them guide our conversation. It was not long before she began to tell the *hadithi*, or story, of Cesarean deliveries, which she often referred to as *operesheni*, or the operation. She first spoke on the cultural implications of her operation. She said “Once a person has undergone the operation our society does not care about us. They want us to give birth, but they do not help us when we have gone through an operation”. She explained that in her culture, women within the community care for each other following delivery, as they must rest until the new child's umbilical stump falls off. Following her Cesarean delivery, though, KN found herself ostracized without support from her community. She told of her young son, who she relied on to care for her while she was unable to do so herself. She summed up her experience saying “if we undergo an operation, [the community] see[s] that we can't do anything. It's like we've become a disabled person…”.

Following this, she wished to share vividly of her most recent birth experience that proceeded these events. Once KN became pregnant, she decided to seek care at a major tertiary care center, where throughout her prenatal care, she expressed a desire for a vaginal delivery. At that time, her care team agreed a trial of labor after Cesarean section (TOLAC) was a reasonable option with her history. However, due to her diagnosis of gestational diabetes, she was advised to be induced at 39 weeks. She initially spoke of her community's hesitance at this, sharing her pastor told her “the problem [in] this country [United States] is that they send children to be born before their time has come”. However, she came to the hospital to be induced at that time, explaining “they said that they will help me give birth naturally. I was relieved and I felt pleasure – I can't explain the joy I had”.

However, as she described her delivery, I learned that her initial fears were realized. Once she began her induction, she told me of what occurred: “Just then, after [saying] I will not be operated on, I fell asleep… Now, after I [woke up]… they told me that the child was breathing badly, and they should do an operation”. She protested, stating again she did not want a Cesarean section, however her medical team proceeded with the Cesarean section. In review of her chart^3^, I discovered consent was appropriate and the Cesarean section was indicated due to fetal bradycardia. However, Cesarean delivery was delayed longer than anticipated, as KN declined the operation for several minutes before consenting.

Despite giving appropriate consent, KN felt harmed by this procedure. She expressed shock, saying, “[I thought], “how will I tell the community?” and “how will I explain to my husband?” … I don't even remember signing the forms. I didn't have someone there to help me fill in the forms”. She felt unheard by her providers, expressing that “[the medical staff] think it is easy, but they don't know the problems we are going through in the community.” She elaborated further to say “because [at the hospital] they saw me as if I was confused, [but] what they did to me violated my human rights”.

When I first heard this, I was shocked. I resisted the impulse to jump to the provider's defense, because I wished to understand, knowing that KN's story held value. Hoping to uncover another perspective, I interviewed the attending physician involved in KN's care. Although consent was eventually given for the indicated Cesarean section for fetal bradycardia, the delay of consent was particularly distressing for the provider. Through the conversation, the moral stress encountered was apparent as she recounted this experience: “I remember in that moment sitting next to her and feeling my heart pounding, doing my best to stay calm in that moment and trying to build connection and trust with her”. The physician realized that during this encounter, her priorities, in order, were a healthy mother, healthy baby, and then vaginal delivery. It became clear that KN had different priorities and a cultural collision occurred in the operating room.

## 3 Discussion

As the population of the U.S. changes and international immigration becomes the main driver of population growth, providers will continue to encounter the many perspectives immigrating populations carry (1). When immigrants and refugees engage with healthcare in their new home, many factors have been cited that drive behavior and perceptions of their care. Cultural norms is one of the more pervasive barriers to healthcare system utilization (9). For each cultural group, this may look different; one example in the literature include Bhutanese-Nepali populations only accessing healthcare for emergencies and illness, rather than for regular preventative visits (10). Another study of parents of Syrian refugee children reported preferring to use traditional healing methods first before engaging with the health system (11). Refugee populations have also cited language and social stigma of disease as other drivers to limiting interaction with the health system (9). Even though the literature is scare of specific cultural conflicts, the importance of understanding the increasing diversity of our patients is clear.

Understanding these differences calls for the practice of cultural humility, rather than cultural competence. Cultural humility involves acknowledging how a person's cultural beliefs affect their behaviors and brings an attitude of continued learning and reflection, where cultural competence expects providers to be well-acquainted with each patient's cultural nuances (12). Critical is the notion that partaking in cultural humility is a life-long learning process, where nonjudgemental curiosity is key to seek information about cultural practices that are encountered (13). In addition to cultural practices, cultural humility also aims for awareness of physician-patient power imbalances that work to place patient narratives at the center of their healthcare (13).

Within obstetrics, patients come with their own priorities for their delivery experiences, which are invariably impacted by different life experiences, cultural expectations and personal perspectives. Women migrating to countries in the Global North may carry differing views on pregnancy and childbirth. For example, East African people view childbearing as a natural process and the Global North perspective as over-medicalizing (14). During my interviews, another participant even told me after Cesarean deliveries, women will often hear their community members say, “This is not a woman”. These cultural views may lead to the refusal of recommended treatment by providers, putting both parties in a difficult position. In the long term, further cultural conflicts can perpetuate the mistrust of the health system for culturally diverse populations within their new home (15). This may then lead to later initiation of prenatal care, which has been shown to increase the risk for preterm labor, low birth weight, and congenital anomalies (15–17). Furthermore, first-generation immigrants who experienced perceived discrimination in their healthcare had a negative association with physical and mental health (18). Each of these outcomes, whether directly related to maternal health or mental well-being, may impact these culturally diverse populations to avoid future care.

The American College of Obstetricians and Gynecologists (ACOG) committee opinion provides guidance on navigating dilemmas when patients decline obstetric intervention during their care (19). These recommendations include engaging in discussions of reasoning behind decision making, evaluating patient understanding of the gravity of each situation, using a multidisciplinary team, and acknowledging the limits that arise for each situation (19). Although the committee opinion exists, difficulties may still arise. Throughout this project, I encountered many providers who reported similar scenarios with women whose cultural views became a point of contention during delivery. Table 1 highlights the ACOG recommendations regarding caring for patients with diverse cultural experiences. Additional commentary specifically addressing how these perspectives may be particularly important for populations with specific pregnancy-associated cultural mandates has been added. Specific commentary on these culturally complex cases involves engaging in conversations about cultural preferences early and often, and the use of interpreter services so that full understanding is conveyed. Further commentary is provided in Table 1.

**Table 1:** Highlighted recommendations from ACOG committee opinion with commentary of cultural implications.

ACOG committee opinion no. 664 (19)	Author commentary
Pregnant patients remain the right to refuse recommended medical or surgical treatment, and those decisions should be respected	–
Use of coercion, manipulation, or threats, including threats to involve courts or child protective services, to sway towards specific decisions are never acceptable. Instead directive counseling should be used, offering advice, guidance and recommendations rather than compelling action as with coercion.	Court-ordered procedures disproportionately affect disadvantaged populations such as women of color and low-income women. Directive counseling still holds that patients may still refuse recommendations and those decisions are to be respected.
Eliciting reasoning, lived experience and values is important in engaging with women who refuse intervention. It is best to understand the context in which a patient is making a decision as there are many areas that hold importance, ranging from religious or cultural grounds, converging interests of multiple parties, misunderstandings, and experiences of family and friends.	When providing treatment for those with cultural backgrounds different from us as providers, the practice of cultural humility may aid in these conversations, allowing for an openness to best understand where the patient is coming from.
There is special consideration to the patient's understanding the gravity of the situation and the risk involved, and the urgency of the case.	For patients that present with a language barrier, there is a need for skilled interpreters to help patients understand. Additionally, inclusion of community leaders who can help clarify certain values and find acceptable areas for compromise can aid with these discussions.
Informed consent process begins before decision making so that the patient is able to make an informed choice.	Conversations on birthing preferences should occur early and often, allowing time for providers and patients to learn from each other and come up with a treatment plan that aligns with patient values. In addition, discussion of potential complications should occur as to not blind-side a patient into a decision. Finally, documentation of these conversations is paramount so that the managing provider is able to be well-informed when encountering this patient.
Differences should be resolved using a team approach, incorporating ethics consultants when necessary in conflict resolution.	An interdisciplinary team approach is a key factor to providing best outcomes. In addition to ethics consultants, experts on specific cultural nuances and skilled interpreters are key team members to keep involved in care.
Acknowledge the limits within decision making: patient understanding of the clinical situation; cultural, social, and value differences; power differentials; and language barriers.	In addition, the urgency and emergent nature of a situation is a limitation. Timing may not allow for full discussion of perspectives or to assess for full understanding. A final limitation may involve the shifting make-up of the medical team where a patient's delivery may not be managed by the provider that knows them best. Additionally, there may be a loss of communication when handing off care from one team to another.

As physicians, it is imperative that we understand how patients' lives outside of the healthcare setting may impact how they engage with us and our teams. However, cross-sectional studies at major medical schools have identified that less than half of fourth-year students feel prepared to deliver cross-cultural care (20, 21). One in four residents felt unprepared working with both patients whose health beliefs were at odds to medical practices in the Global North and new immigrants to the United States (22). Many medical schools have attempted to incorporate cross-cultural education into their training, taking both formal and informal forms. However, half of residents reported receiving little to no cross-cultural education after medical school (22). Contrast this to a survey of healthcare professionals who were confident of their ability to meet the needs of their multi-cultural group of patients (23). However, their confidence came from awareness of cultural norms and using interpreters. Systemic cross-cultural components, such as racism and historical power imbalances were not acknowledged (23). This underlines the importance of incorporation of both skill-focused and systemic education for medical trainees and current providers. To this end, it also suggests the need to engage in the practice of cultural humility (12).

Incorporation of cultural humility and cross-cultural education into medical training has been recognized by others outside of just the field of obstetrics. For dermatology, this has taken many forms, such as developing educational programs to increase cultural awareness and recognition of skin disorders in people of color (24). They have also recognized the importance of a culturally diverse field of trainees and providers that allow for collaboration and peer-to-peer learning (24). For emergency medicine residents, a recent study showed that case-based, near-peer teaching on health disparities facing vulnerable cross-cultural patient populations improved resident self-reported cultural humility (25). These trainees reported increased awareness of cultural differences and that their care approach for these marginalized groups changed (25). Similar cross-cultural education has been incorporated into the training of surgical, psychiatric and pediatric residents with improvements in cross-cultural approaches to care (26–29).

Cultural humility can also serve the purpose of addressing both explicit and implicit bias carried by healthcare providers. By turning their lens from being experts in these situations to a position of curiosity through cultural humility, physicians can work to acknowledge their biases (30). Following a course that provided teaching on self-reflection and culture in these settings, students reported feeling more aware of their biases and an increased commitment to continued learning throughout their careers (31). Another important facet for these cross-cultural population is the framework of intersectionality, broadly defined as how the intersection of individual identities, social status, power and systemic practices impact how individuals are understood and treated in society (32). Applying this framework for the participants in this study allows us to see multiple intersecting and interrelated identities of these women, such as mother, wife, woman, community member, church-goer, and immigrant. Each of these identities impacted the way these women engaged with the health system, and their health beliefs. By recognizing the intersectionality for patients, it can help providers practice cultural humility, leading to more questions and non-judgmental approaches to better understand each patient, their beliefs and their health needs (33).

With these approaches in mind, I suggest implementation of several points to improve cultural education for residents that stretch beyond the specialty of obstetrics and gynecology. Firstly, the implementation of didactic lectures during protected learning time to address cross-cultural communication skills is an important factor. Additionally, formal learning experiences to understand the history of the social landscape of the program's geographical area would give context to patient population residents care for daily. The topics of these presentations could model the near-peer didactics presented in Tsuchida et al. and should be tailored to fit the needs of the specific specialty (25). Through the lens of Ob/Gyn training, this may include cultural preferences in obstetric care that providers navigate with their patients in their delivery. Through these didactics, I suggest the continued practice of cultural humility, which may include cultural awareness training, engaging in self-reflection, and working to overcome language barriers (12).

Outside of formal didactic time, allowing residents to work with culturally diverse populations will give experience needed to care for these patients in the future. Focused feedback should be provided to residents on their ability to communicate with patients in a cross-cultural setting with the goal of continued improvement. Finally, it is important to note 58% of residents reported lack of time to be a problem for them to deliver cross-cultural care (22). For this, structural adjustments to allow for extended time slots at continuity clinics for multidisciplinary care should be placed, acknowledging that many patients require additional time for navigating language barriers and providing culturally humble care (12).

For myself, I find that through KN's story and this project, cultural humility has been emphasized, and in some ways, I have also been engaged in the practice myself. Reflecting on the process, I understand that as a cisgender white American male, I will never be able to fully understand the perspective of the women with whom I spoke. I will never become pregnant; I carry privilege as a white man in our society; and I understand American medical culture. I also acknowledge that the practice of cultural humility is a life-long process and will be of continued importance in each patient interaction. While I am nearing the end of my medical school years, I hope to continue to carry these stories as I work to become the best provider I can be for my future patients.

## Data Availability

Due to ethical and privacy considerations related to the small sample size, the raw interview data cannot be shared. However, a redacted version of the dataset, ensuring participant confidentiality, will be made available by the authors upon reasonable request.

## Footnotes

^1^Although conversational in Swahili, interviews were conducted with an interpreter present to aid in my understanding. Interviews were recorded and transcribed with the aide of the same interpreter.

^2^The study that inspired this perspective piece was approved by the University of Kansas Medical Center's Institutional Review Board. KN (alias for the participant) consented to the sharing of this story.

^3^KN was consented for chart review following this interview as part of the project.
